# Transactions of the Medical Society of London

**Published:** 1817-06

**Authors:** 


### PART II.

### COMPREHENSIVE ANALYTICAL REVIEW OF MEDICAL LITERATURE.

"Tros, tyriusve, nobis nullo discrimine agetur."

*Transactions of the Medical Society of London. Vol. I. Part 2.* Octavo, pp. 320. London, 1817.

This Part consists of twenty-three Articles, many of which are very interesting. Upon the whole, without being rendered so expensive by plates as the volumes of the Medico-Chirurgical Transactions, the work before us, as far as it goes, may fairly stand in competition with the generality of its contemporaries, and it furnishes a very creditable specimen of the talent of this Society. The first half of the volume was published long ago, and was reviewed in the first volume of the New Medical and Physical Journal.

### Art. 1. *On Epilepsy.* By Dr. Adams.

This is a long, but by no means uninteresting Article. Dr. Adams has displayed considerable ingenuity in the theoretical part of the paper, and what is of more consequence, has evinced much talent in his therapeutic views. He divides Epilepsy into acute and chronic. Under the former head he describes the well known paroxysms of this dreadful disease, preceded sometimes by higher health than ordinary; at others, for a few days, by heaviness and an incapacity in the patient to arrange the objects of his attention with the usual accuracy. These symptoms will often cease a few hours before the attack, and be succeeded by an hilarity which, though part of the disease, will, by the friends, be considered as the cause. The paroxysm itself, which, at first, generally takes place in the night, is characterized by insensibility; uncertain duration; universal convulsions; præternatural power in the muscles; involuntary discharges; redness of the skin; turgescence of the external veins; foaming at the mouth, and the various phænomena which are usually observable in violent seizures of the epileptic kind.

The paroxysm is succeeded by a profound sleep, from which the patient awakes, wild, and unconscious of all that has happened. Sometimes he continues for a few days deprived of reason; at others, nearly in a state of idiotcy painful to the beholder. From these he gradually recovers; and continues well till the next seizure, which generally returns at regular periods, of from three to six weeks. If these be frequent and violent, the patient has not time to recover his intellects between them; at length he becomes idiotic or maniacal; or dies apoplectic.

Dr. Adams informs us, that *post mortem* examinations have, in these cases, discovered, 1st. where the patient dies of any other complaint, after several acute epileptic attacks, a firmer and more elastic texture of the brain; with the cavities somewhat larger. 2d. In death during a paroxysm, a considerable quantity of fluid is found in the ventricles of the brain, which is still firmer, with adhesions of the pia mater, and frequently layers of coagulable lymph. 3d. If death take place at an uncertain period after the paroxysm, during which interval there is aberration of reason, or alteration of temper, we usually find suppuration in the brain. Without pausing on the history and etymology of this terrible disease, in which Dr. A. displays his usual acuteness and research, we shall proceed at once to state our author's pathology in his own words.

"On a review of all the symptoms of acute epilepsy and of the appearances after death, I have no scruple in referring the whole to inflammation of the substance of the brain. No one is ignorant that in many epileptic subjects no change has been observed in the appearance of that organ; there are, however, many authorities on the contrary side. It does not, therefore, seem unfair to impute this difference to the different forms of a disease, too often included under one general term." p. 11.

This inflammation, Dr. A. thinks is of the adhesive kind, and he endeavours to account for the *ratio symptomatum* in the following way.

"The previous heaviness of the patient is the effect of plethora, which has rendered the vessels of the brain so full as to require the adhesive inflammation to support them. Inflammation is increased action. It is, therefore, attended with increased powers, or a greater capacity of bringing original powers into action; hence, the increased vigour and hilarity which sometimes immediately precede a paroxysm. The same has been remarked by Sydenham, immediately previous to a gouty paroxysm, and is among the first aphorisms of Hippocrates relative to disease in general. But when inflammation is very considerable in any organ, the customary functions of that organ are suspended. This is well known in the intestinal canal, and in the kidnies. Even the muscles cease to act under violent inflammation, an effect extended to the muscles of the heart itself. From the cessation of the usual functions of the brain, we should expect a total insensibility to every impression on the organs of sense, and an entire extinction of memory, and such is actually the case as long as the high inflammation or epileptic paroxysm continues. But the muscles are put in action by stimuli and sympathy, without any consciousness of the mind. Hence, the great irregularity of their convulsive contractions, when they are no longer influenced by the mind, and their increased violence in contracting, probably, from the strong impressions conveyed through the nerves from parts of the brain under the increased action of inflammation. This violent action is, as in all other cases, succeeded by torpor, during which the patient is, or appears asleep. The rigidity of the muscles for a few days afterwards is the necessary consequence of their violent exertions, and the dulness of the senses is the effect of the effused lymph. As the lymph is absorbed, the functions of the parts are restored; thus the patient returns to his memory and reason in a greater or less time, in proportion to the violence and length of the paroxysm, and the consequent larger effusion of lymph. Sometimes the quantity of lymph effused is so considerable, that the whole is not absorbed before the periodical return of the paroxysm; in this case a fresh effusion takes place, and the quantity of adhesion in the substance, and of fluid in the ventricles of the brain, is thus gradually accumulated, till all memory is lost and the patient becomes idiotic. If suppuration has taken place the symptoms subside in part, but the patient is rarely perfectly restored." p. 16.

Although we think that we could bring many and strong objections to the foregoing hypothesis; yet, as it is at least as good as those hypotheses which have preceded it, we shall leave it to the consideration of our readers. To Dr. Adams's curative views we cannot possibly object, as we believe them correct, as far as they go. During the paroxysm, he thinks that little can be done; unless the symptoms are violent and long continued, when it is absolutely incumbent to bleed freely in order to prevent extravasation, suppuration, or high inflammation. The patient and friends should be taught the probability of a succeeding paroxysm in a few weeks; and by way of precaution, in about three weeks it will be right to bleed him to eight, ten, or twelve ounces, according to age or other circumstances. To this precautionary measure must be added other evacutions, particularly from the bowels, and a reduced diet. Dr. A. has not seen any unusual degree of plethora or other kind of inconvenience follow periodical venæsections for this complaint; and we believe such apprehensions are in general chimerical.

The precise limits between acute and chronic epilepsy, he acknowledges, are difficultly fixed. But he thinks that in the latter, the periods are less regular, and are sometimes brought on by mental associations. He does not believe chronic epilepsy is caused by inflammation of the brain, since—"None of the symptoms subsequent to inflammation of the brain, appear, after a slight paroxysm, nor any change in the appearance of the brain of those who have, for many years suffered from chronic epilepsy." Dr. Adams thinks, that this species of epilepsy is absolutely beyond the control of medicinal agents.

"But if," says he, "we have no remedy on which we can depend for chronic epilepsy, it is one consolation to add, that the acute form, which alone threatens life or intellect, has, *in every well marked case* that has occurred to me, been cured in the manner above described." p. 35.

We believe we could produce proofs, that the acute species is not so tractable as Dr. Adams has found it, and that the chronic species is often under the control of counter-irritants, internal and external; particularly the lytta. As, however, he appears to have imbibed the scepticism of Heberden on this point, we cannot expect to alter his tenets by any thing that we can say.

### ART. 2. *On the Treatment of Croup.* By HENRY BLEGBOROUGH, Esq.

MR. BLEGBOROUGH having, in several Cases of Croup, employed without success, the usual antiphlogistic means, as bleeding, purging, nitre, digitalis, blisters, &c. he resolved to try nauseating doses of the tartrite of antimony, as a powerful mean of lowering the tone of the circulation, and arresting the progress of inflammation.

The first case was unsuccessful; but while the antimony was persevered in, "the complaint made no perceptible progress." It was left off at the request of the parents and another practitioner, and the child died.

*Case* 2. A fine boy, four years of age, was seized with Croup. Mr. B. abstracted four ounces of blood, prescribed ten drops of tinct. digit. every four hours with two grains of calomel: A blister to the neck. Second day, no alteration; no stool; continue the medicines. Third day, has taken twenty-eight grains of calomel; and ninety drops of digitalis. Breathing considerably relieved; cough less harsh; skin cool; has passed two dark green stools. *Evening.* Has taken a scruple more of calomel; one stool; croupy symptoms disappeared. An expectoration succeeded, and the child recovered.

*Case* 3. We shall give this in our author's own words, as it will afford a fair specimen of his mode of treatment in croup.

"*November* 22, 1808. At three, P. M. I was called to see John Ward, of Curtain Road, a tall, thin, weakly boy, of six years, who was suddenly seized in the morning of that day, with frequent, hoarse, and violent cough, long and laborious inspiration, with great sense of narrowing of the glottis, fever and thirst; tongue clean; pulse 130, but not very full; bowels regular. I took from his arm three ounces of blood, when he fainted. I directed him calomel gr. ij omn. hor.; to drink toast and water; diet, gruel only. November 23, eleven, A. M. has taken thirty grains of calomel, cough and difficulty of breathing the same; voice almost lost; noise on inspiration great; blood does not exhibit buff; heat considerable; tongue covered; pulse 138; no stool. Continue calomel. 24th, eleven, A. M. has taken thirty-six grains more calomel; at six this morning crouping suddenly ceased, cough frequent, difficulty of breathing much diminished; countenance cheerful, pulse 128, tongue moist; has had two stools of a dark green colour; discontinue calomel: haust. purg. 25th. Draught operated powerfully, cough less frequent without much expectoration, breathing good, pulse 120; tongue moist, skin cool, appetite moderate, thirst abated. 28th, has expectorated a good deal of a substance resembling a mixture of pus and curd, which still he continues to do, though in less quantity; in other respects he is well." p. 44.

Several other cases are related by Mr. B. most of which were successfully treated on the foregoing principles, and therefore we deem it not necessary to dwell farther on this article. We believe the plan of cure here recommended, is that generally pursued in this very fatal infantile disease; but we fear not with similar success. The warm bath, and early blisters to the feet or legs would be useful auxiliaries to the above plan.

ART. 3. A long Article on the same subject follows from the pen of William Gaitskell, Esq. from which we find little to extract. Mr. G. asserts, contrary to the opinion of Mr. Blegborough, that he never remembers to have witnessed one case of croup of more than twelve hours' duration, ending favourably under any treatment.

He distinguishes the disease into idiopathic, symptomatic, and venereal; but confines his remarks to the former species.

"Reason and experience," says he, "shew, that the only prospect of success must be from copious depletion of the vessels, with counter-irritations in the neighbourhood of the part, and the promotion of the natural secretion. The indications are best fulfilled by bleeding, vomiting, blisters, the warm bath, and purging." 73.

I. *Bleeding*, to be employed early, from the jugular or brachial veins. "*Should syncope follow, the symptoms will immediately cease.*" Leeches to the superior part of the sternum, rather than to the larynx, because pressure may be used to stop inordinate hæmorrhage. When the actions of the system are reduced, expectorants, especially the tartrite of antimony, till vomiting is excited.

II. *Blisters*, after the violence of the disease has been mitigated by general and local bleeding:—To be applied to the sides of the throat; nape of the neck; or upper part of the sternum.

III. *The Warm Bath*, at 96, *after* bleeding and vomiting. Pediluvium.

IV. *Purging*, especially with calomel. Within these four months, Mr. G. saw three cases of genuine idiopathic croup yield to bleeding, vomiting, blistering on the breast, pediluvia, and purging with calomel.

"The children took two grains of calomel every two hours till dark coloured stools were procured."

The calomel was then continued every four hours, in the same doses; more stools were furnished, and in forty-eight hours the disease terminated favourably. In the Spring of 1800, Mr. Joseph Gaitskell treated two cases with calomel alone.

"These children were brothers, the elder four years old, the younger two, and were attacked at the same moment of time. The elder took three grains of calomel every two hours, the younger two grains; when thirty-six grains were taken by the former, and twenty-four by the latter, some dark green stools were evacuated, with considerable alleviation of the symptoms. By the time ten grains more were taken by the elder and five by the younger, the disease was subdued and required no fnrther use of medicine." p. 82.

Mr. G. considers that in the idiopathic croup of infants, bronchotomy would be unavailing from the small calibre of the trachea. But in the *venereal* croup of adults he thinks it deserving of trial. He has seen five cases of this disease, two recovered under mercurial frictions and cinnabar fumigations; the other three died suffocated. In this species there is no membrane produced; but a venereal ulceration in the sacculus laryngis, or on its border, which, by the secretion of pus, and excessive irritation of the muscles of the glottis, generally terminates life. To allow time therefore for the introduction of mercury, bronchotomy promises success. Mr. H. once performed the operation on a woman thus circumstanced, and she lived six days, with every hope of final success; but was suffocated by neglect of the nurses.

ART. 4. *Three Cases of extraordinary periodical Sickness; two of which were cured by Arsenic.* By JOSEPH ADAMS, M. D.

I. A young gentleman had for many years been afflicted with a ten-day periodical sickness, which lasted, each time, several hours, and left him well during the intermediate nine days. Dr. Adams conceiving the complaint to be a species of intermittent, prescribed Fowler's solution, which soon broke the morbid catenation, and relieved the patient from his disagreeable visitor.

II. A married lady had been troubled during fourteen years with a periodical sickness, which returned precisely on the same day of every week. The nausea was so distressing, that she was obliged to drink warm water during the greater part of the day, which, by bringing up some mucus, gave her momentary relief. She had borne a healthy child every year; but there was no alteration produced on the complaint by gestation, parturition, or lactation. She was habitually costive. Dr. A. prescribed a brisk purgative, and subsequently five drops of Fowler's solution thrice a-day, which dose was slowly increased. The disease was soon cured.

III. This was a Dispensary Case. The woman affirmed, that she had been for eighteen years affected with a weekly periodical sickness, attended with frequent headache at uncertain periods. The sickness yielded to the arsenic; but the head-aches remained. From irregularity, &c. the case is not considered conclusive evidence by the author.

We every day remove obscure morbid phænomena, constant as well as periodical, by this valuable medicine. Indeed its use is much too limited in medical practice; and its bad effects greatly exaggerated.

### Art. 5. *An ounce of Sulphuric Acid swallowed.* By Joseph Adams, M. D.

In the month of April 1813, a woman swallowed about an ounce of sulphuric acid, rendered black by the cork, in a mistake for some physic. While her husband ran for medical assistance, the patient swallowed all the water that came within her reach. The surgeon on his arrival found her vomiting most violently a black liquid. An antimonial emetic, and afterwards ol. olivæ, with warm water and kali purum. Although the vomiting continued incessant, the woman never perceived that the acid returned; but only a black fluid proportioned to the thin liquids she drank. No appearance of excoriation or inflammation in the mouth; no medicine would stay on the stomach or appeared to give any relief. About twelve days after the accident, she was seized with a more than usually violent paroxysm of sickness, whilst the surgeon, [Mr. Aldridge] was in her chamber. As he held the basin, he heard her exclaim, "Oh, the vitriol is come up!" This was considered to be merely "in consequence of her vomiting being *more acid than usual.*" But Mr. A. having observed afterwards a brown spot on the sleeve of his coat, and in the course of a few days a hole, equal to the spot, and several smaller ones in different parts of his dress, Dr. Adams and himself came to the conclusion that the sulp. acid had remained enveloped in mucus till that time. The woman, however, experienced very little relief from this disgorgement of the poison, "the sickness remaining nearly as distressing as before," nor did any thing but *time* alleviate her afflictions. She perfectly recovered in the end. Although we are forced to rank ourselves among the sceptics on this occasion, we shall allow Dr. Adams to explain in his own words.

"The vitriol was swallowed early in the morning, before any kind of food or drink had been taken to wipe off or dilute the mucus of the mouth, throat, or œsophagus. When received into the stomach it would coagulate the mucus with which it came into contact, and stimulate the stomach to secrete more. By these means it might have been contained in a number of cysts of coagulated mucus, or in one or two large cysts which might have been thrown up at the time Mr. Aldrige perceived the stain and consequent hole in his coat. At the same time, it can hardly be expected that these cysts would be sufficient entirely to preserve all the acid from contact with the stomach, which being denuded of its proper mucus, might be inflamed to a certain degree, and thus secrete a substance consisting partly of blood, which when thrown from the stomach, has frequently this black appearance." p. 101.

**Art. 6.** *Lusus Naturæ of the Female Organs of Generation.* By W. Gaitskell, Surgeon.

Mrs. S. ætat. 22, married, had never menstruated, and conceiving that there was something wrong, submitted to examination.

"On separating the labia pudendi, the clitoris was discovered, with its preputium: the meatus urinarius and nymphæ in a perfect state. Between the nymphæ from the inferior edge of the orifice of the urethra to the fourchette, there was a pale red corrugated membrane, which I judged to be hymen; but, on accurate investigation, it proved to be vagina, which, when unfolded by the finger, at two inches from its entrance, terminated in a cul de sac."

On examination by the rectum, the uterus could not be felt; and hence surgeon Gaitskell sagaciously concludes, that "the uterus was entirely wanting; but probably some of the uterine appendages present and efficient." 105. We do not consider the phænomena from which Mr. G. drew this conclusion as at all authorising it; nor can we see the great importance of the conclusion, if it were correct.

**Art. 7.** *Case of Vermis Lumbricus perforating the Intestinal Canal and Abdomen.* By J. C. Lettsom, M. D.

Eliz. Gwillim, ætat. 15, consulted the Doctor in June, 1810. She had hectic symptoms, which gradually induced emaciation of all parts except the abdomen, which was large and tense, although diarrhœa was troublesome. Dr. L. considered the tumescence and hardness as resulting from enlargement of the mesenteric glands. Leeches were applied to the abdomen, with the fotus papaveris. Pil hyd. given internally, with sulph. sodæ in infus. rosæ. The cough and fever gradually subsided, and the abdomen was reduced. In July, a circumscribed inflamed tumour appeared a little below the navel, about the size of a crown piece, and suppurated. When opened, a puff of air followed the matter, but no fæces. On the 8th of August, a large lumbricus teres was discharged *per anum*, and the patient's health progressively improved. On the 12th of August, on removing the poultice, a lumbricus presented, and was carefully drawn out. It was alive, and measured nine inches in length. Small air bubbles occasionally appeared at the orifice, as well as some fæculent matter—both ceased in a few days. Some seeds of apples or pears which she had eaten, occasionally protruded. She perfectly recovered.

Dr. Lettsom speaks highly of *ol. terebinth* in the expulsion of ascarides that tenaciously infest the lower portion of the intestinal canal. It also appears by a letter from Dr. Walker of Leeds, that the latter gentleman was the first who exhibited ol. tereb. in worm cases, viz. in the year 1798.

Art. 8. *Diseased Action of the Heart, &c. effectually relieved by Blood-letting and the horizontal Posture.* By H. Clutterbuck, M. D.

This is a most important and interesting case, and we shall consequently give a full analysis of it.

Mrs. C. ætat. 35, married, applied to Dr. C. *October 20th,* 1814. General appearance exceedingly distressing; countenance expressive of great anxiety; skin perfectly pallid and ex-sanguine, except lips and cheeks, which bore a leaden hue; tongue clean, moist; ex-sanguine; extremities cold; pulse weak and irregular; breathing much oppressed; face bloated; legs œdematous to the knees; constant uneasiness in the region of the heart; frequent palpitations, even when in bed; always upon walking; pulse at the wrist irregular during palpitation. Upon making any unusual exertion, the cardiac uneasiness is aggravated to the degree of acute pain, extending to the back, collar bones, and middle of the upper arms, particularly the left. Menses regular in period, but trifling in quantity, and nearly colourless. Appearance altogether chlorotic, with not a few symptoms of hydrothorax, or hydro-pericardium. Appetite very bad; great uneasiness after eating; constant constipation; general strength greatly reduced; apparently in a very dangerous state, of which she herself was sufficiently aware.

These symptoms had continued for several months, gradually increasing. They commenced soon after an inflammation in the chest.

*October* 21. On visiting her to day at her own house, the violence of the symptoms were found much abated, from her being at rest and in bed; the pulse was tolerably regular.

"The symptoms above described sufficiently indicated an excess of irritability and disordered action in the heart; while their duration, severity, and their having succeeded an attack of inflammation in the chest, gave reason to apprehend, that the disposition to such irregular action in the heart was the consequence of some disorganization having taken place." 124.

As it was to be feared that inflammatory action was still going on, there did not appear any means of checking it so probable as venæsection, notwithstanding the debility and seemingly bloodless state of the patient. About five ounces were cautiously abstracted from the arm, which she bore without inconvenience. The crassamentum was in proportion, and somewhat cupped, but without buff. It presented a leaden hue. Evident relief was experienced by the loss of blood, and thus the road to further advantage seemed pointed out. *Digitalis* was administered in small and frequent doses, as was *ammonia,* with the view of exciting a little action in the stomach, and of determining to the surface. Several evacuations by stool were daily procured by aperients. Plain, easily digested food was allowed. All strong drinks were prohibited, "and above all things, quiet of body and mind, and a horizontal position were enjoined." She was confined to bed almost entirely for ten weeks. The blood-letting was repeated at intervals, and the plan altogether persisted in, with great regularity, for nearly three months, with gradual and continued amendment; and at the end of this period, her health was perfectly restored. She had lost every uneasy feeling about the chest; the pulse became quite regular; the œdema of the extremities disappeared; appetite returned; the bowels acted readily; menses returned; and the complexion became natural. She was bled, in the whole, four times from the arm, and once by cupping from the nape of the neck. Whenever the heart acted irregularly, a troublesome beating was felt in the head; this yielded with the other symptoms.

Dr. Clutterbuck justly observes, that this case shews that symptoms of an alarming nature, apparently indicative of organic disease of the heart, or at least threatning disorganization, are not altogether hopeless. A case which seemed of all others, from general appearances, to be most unfit for blood-letting, not only bore this evacuation with impunity, but was effectually relieved by it.

"And if so, (says this excellent physician) it appears further to be probable, that the employment of remedies of an opposite nature, such as tonics and stimulants, which are generally had recourse to in such cases with a chlorotic character, and with swelled extremities, would not only have proved unavailing, but in all probability have aggravated the disease." 128.

### Art. 9. *Dyspnœa not satisfactorily accounted for on Dissection.* By Dr. Lettsom.

Eliza D——, ætat. 22, early in May, to avoid a storm, walked very fast, in which exertion she felt an uneasy sensation in the breast, as if something had suddenly snapped. She found immediate difficulty of breathing, which continued more or less for three weeks, till Dr. L.

saw her. At this time her breathing was laborious, quick, with such palpitation of the heart as was visible across the chamber; oppression rather than pain about the præcordia. Pulse 140, feeble, occasionally intermitting; lay on both sides equally well, and with the head low. *Treatment.* Blister to the breast; camphor julep, with æther, and small doses of digitalis. Urine at first turbid and scanty; but under the above treatment, it increased and became clear. This was followed by a temporary mitigation of the complaint; but the extreme weakness, palpitation, and rapidity of pulse resumed their former alarming state; but still without delirium, or decubitus difficilis. In the second week after the doctor's attendance the patient expired under increased debility and palpitation.

*Sectio Cadaveris.* Lungs sound; a pint of limpid fluid in the left bag of the pleura; two ounces of water in the pericardium; heart and its appendages sound. Dr. L. says, that he does not recollect any instance of hydrothorax *to such an extent,* wherein the facility of reclining low in bed, and on either side, remained. We refer to page 452 of our first volume, where Dr. Johnson relates a case, in which the patient "could lie on either side, and with the head low," yet *nine pints* of water were found in the left cavity of the chest.

ART. 10. *Case of Strangulated Hernia, cured by Elaterium.* By Dr. WOODFORDE.

THIS is a prolix article, but will not detain us long. We believe that few people who have any regard for their surgical reputation, would trust to any remedy but the knife in a case of strangulated hernia, the patient vomiting fæces, and without a stool for thirteen days. But we are of opinion that there was no strangulated hernia in the case, for these reasons. 1st. Because on the day after the patient commenced the elaterium he had a stool; *the hernia unreduced.* 2d. Because *four days* after this period, (during which he had been constantly taking the elaterium) viz. on the 19th, he had "three copious stools after taking ten of the pills"—*the hernia still unreduced!* But lo! in the afternoon of this day, the hernia began to move of its own accord, and the patient reduced it himself. It is unnecessary to say more on this subject. Had a Cooper, an Abernethy, or a Lawrence sat in judgement on this cure of hernia by elaterium, it would never have seen the light, except as a case of common *ileus.*

Art. 11. *Case of Apoplexy.* By the same.

This was a severe and obstinate case of apoplexy in a plethoric female, ultimately subdued by perseverng evacuations of all kinds. We hope and believe that Dr. Woodforde is mistaken in supposing "that practitioners are become extremely sceptical in the utility of the operation [bleeding] in this disease, notwithstanding the phantoms of debility that haunt some of their minds in other complaints. Dr. W. refers to Morgagni in support of his depletory measures. His letters on this subject have been so recently commented on in our pages, that we need not agitate the question in this place.

Art. 12. *Ill Effects arising from Moisture in Houses.* By Dr. Lettsom.

Some of Dr. Lettsom's pathological opinions are certainly most curiously expressed. Thus, after exposure to moisture "there ensues such a powerful *disorganization* of muscular and nervous *energy* as destroys *vital action*, and terminates existence." Some cases are related where motion was rendered almost impracticable by painful spasms. In one case, these spasms gradually extended to the muscles of the face, exhibiting a similitude to *risus sardonicus* on endeavouring to speak, and occasioning at the same time, considerable difficulty of articulation, the mental powers remaining uninjured. At length the spasms extended over the whole trunk, and threw it into a state of opisthotonos, with impeded respiration. The legs now were more plastic; the arms became enervated, and totally paralytic; the muscles of respiration becoming similarly situated, and nervous energy subsiding, the patient ceased to exist. The warm bath; ammonia; blisters; sinapisms; fomentations and embrocations, were ineffectual; and opiates gave no relief.

We shall terminate the analysis of this interesting half volume in our next number. In the mean time, from the specimens which we have exhibited of the component parts of this work, our readers will, we trust, have formed as favourable an opinion of its merits as we do; and we conscientiously recommend it therefore to be placed on the same shelf with the most valuable depositaries of modern medical knowledge.

